# Supplementary material for: The Role of Early Procalcitonin Determination in the Emergency Departiment in Adults Hospitalized with Fever
Source: Medicina (Kaunas). 2021 Feb 19;57(2):179. doi: 10.3390/medicina57020179 (PMC7922631; doi:10.3390/medicina57020179)

Supplementary Materials

Chart review methodology

The retrospective analysis was made according to best practice in chart review. All data were extracted from the computerized clinical records of our institution by an informatics research. The research was limited to the study period (2009-2018), and extracted all the records of hospitalized adult which accessed the Emergency Department with fever among the major complaints.

A total of 14697 records were extracted.

Based on the study protocol, a pre-definite set of variables was created and data were extracted from the selected records.

Physicians with at least 3 year of clinical experience in ED (MM, AG, MLB, BS, MCan, VO, GdM) revised the clinical records in the period from January to June 2019.

Among the selected records 2635 were excluded. 2185 patients had HIV infection, acute leukemia or lymphoma, 389 patients were on immunosuppressive therapy due to transplant. Finally, 61 records were excluded for incomplete or inconsistent clinical records.

A sample of 50 records was randomly extracted to assess the inter-operator reproducibility of data extraction.

Data integrity and conservation was guaranteed by MCov, FF and RM

Supplementary Figure 1 – Distribution of PCT determinations and controls during the study period.

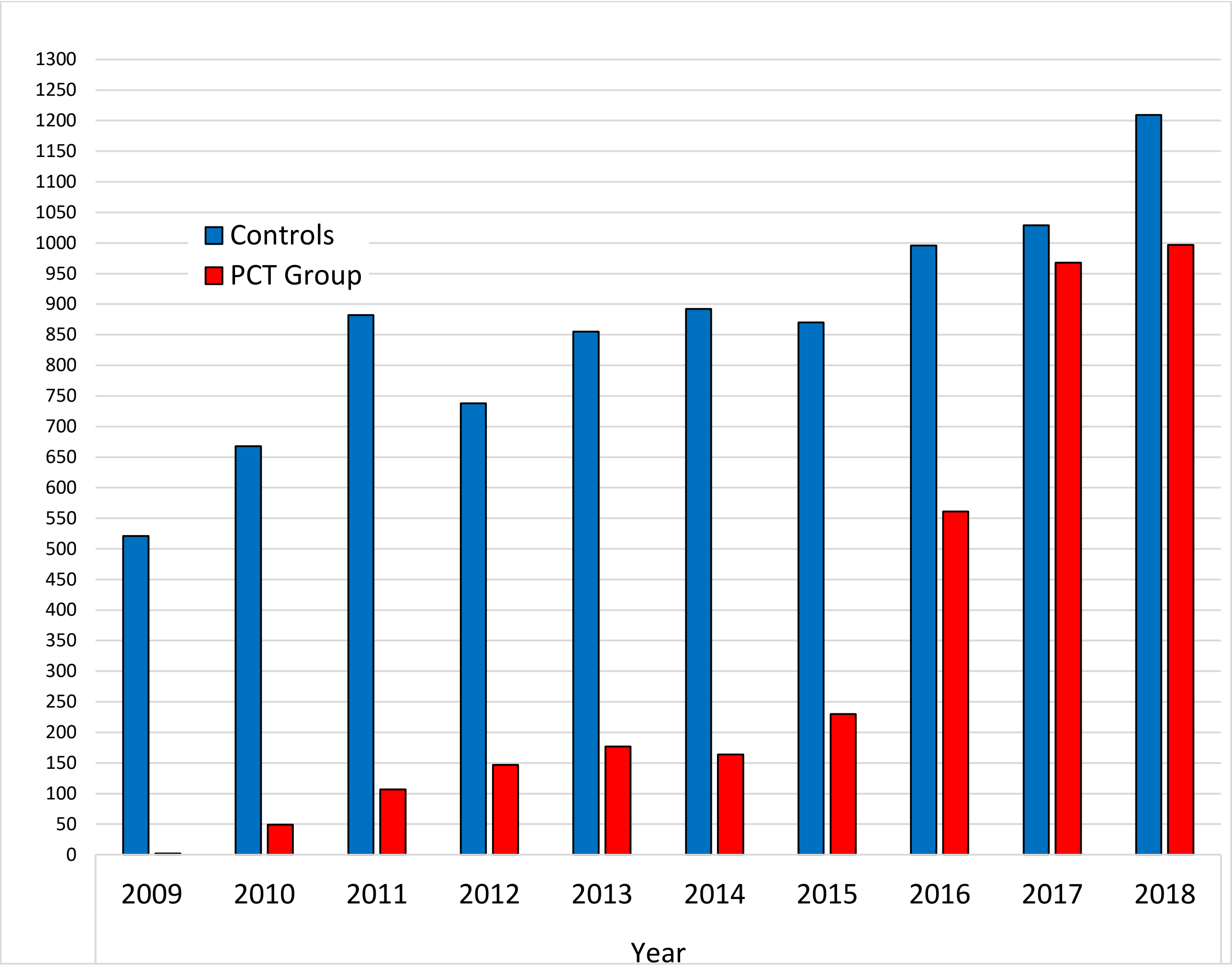

Supplement: Supplementary file 1 [file medicina-57-00179-s001.pdf]
